# Chimeric Antigen Receptor T Cells Targeting CD19 and Ibrutinib for Chronic Lymphocytic Leukemia

**DOI:** 10.1097/HS9.0000000000000174

**Published:** 2019-02-08

**Authors:** Julio Delgado, Miguel Caballero-Baños, Valentín Ortiz-Maldonado, Maria Castellà, Laura Magnano, Manel Juan, Álvaro Urbano-Ispizua

**Affiliations:** 1 Department of Hematology, Hospital Clínic, IDIBAPS, Barcelona, Spain; 2 CIBERONC, Barcelona, Spain; 3 Department of Immunology, Hospital Clínic, IDIBAPS, Barcelona, Spain; 4 Hematopathology Unit, Hospital Clínic, IDIBAPS, Barcelona, Spain

The outcome of patients with chronic lymphocytic leukemia (CLL) has significantly improved with the advent of targeted therapies including rituximab, obinutuzumab, ibrutinib, idelalisib, or venetoclax. Unfortunately, the prognosis of patients who fail these novel agents remains poor.^1^ Chimeric antigen receptor T-cell therapy targeting CD19 (CART19) has revolutionized the treatment of B-cell malignancies.^2^ Around 50% to 70% of patients with CLL respond to this therapeutic modality, with complete response rates around 20% to 25%.^2,3^ These results are, however, worse that those obtained in acute lymphoblastic leukemia, both in terms of response rate and duration of response. Ibrutinib is a Bruton tyrosine kinase inhibitor approved for CLL therapy.^1^ Ibrutinib also inhibits other kinases, including the interleukin-2-inducible T-cell kinase (ITK) and improves T-cell function and preclinical CART19 efficacy.^4,5^ Indeed, a clinical trial is currently evaluating the role of prolonged exposure to ibrutinib before and after CART19 therapy.^6^

At Hospital Clinic we have developed our own CART19 construct (A3B1:CD8:41BB:CD3z or ARI-0001) for patients with relapsed/refractory B-cell malignancies (CART19-BE-01 clinical trial, NCT03144583).^7^ Here we report the outcome of the first patient with CLL treated with ARI-0001 cells.

A 53-year-old woman was referred to us for ARI-0001 cell therapy. She had been diagnosed with Rai stage 0 CLL in 2010. Tumor cells harbored unmutated *IGHV* genes and a heterozygous 13q deletion. In 2011, the patient developed progressive lymphadenopathy and received 6 courses of fludarabine, cyclophosphamide, and rituximab, achieving a complete remission (CR) with negative minimal residual disease (MRD). In 2015, she received second-line treatment with bendamustine plus rituximab, achieving a short-lived, partial response. In June 2016, she was commenced on, and responded to, ibrutinib (420 mg daily), but the medication had to be stopped 8 months later due to persistent grade 2 diarrhea. In February 2017, she was commenced on venetoclax, but developed grade 3 diarrhea even at a low dose (200 mg/d), requiring oral dexamethasone to tolerate the drug. Venetoclax was eventually stopped 6 months later. Idelalisib (150 mg twice daily) was started in August 2017, but stopped 10 days later due to grade 4 diarrhea. In September 2017, she received obinutuzumab monotherapy (1000 mg) but developed progressive lymphadenopathy.

**Figure 1:**
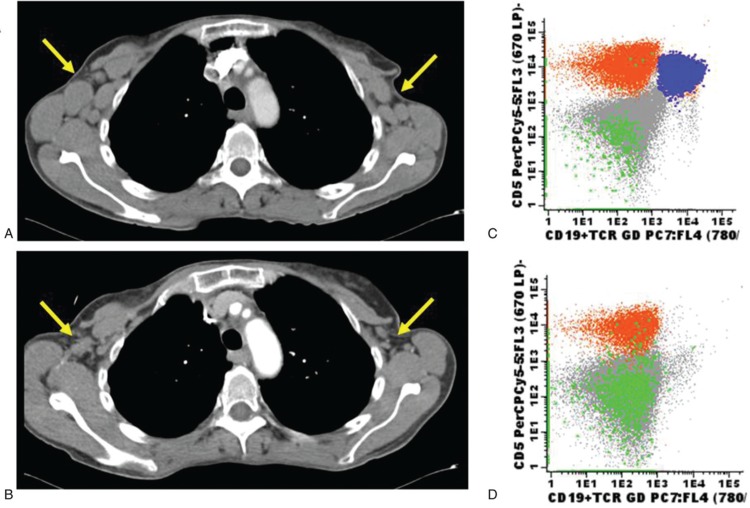
**Panels A and B show a chest computed tomography from the patient before (A) and after (B) ARI-0001 infusion.** Yellow arrows point to bilateral axillary lymphadenopathy remarkably improving upon therapy. Panels C and D show flow cytometry plots from bone marrow aspirates before (C) and after (D) ARI-0001 infusion. CLL cells are depicted in blue while normal T and NK-cells are depicted in green and yellow, respectively. Notice that the patient had received the anti-CD20 monoclonal antibody obinutuzumab right before study inclusion, thus explaining the lack of normal (CD19^+^/CD5^−^) B cells even before ARI-0001 infusion. CLL = chronic lymphocytic leukemia.

In October 2017, she was recruited into the CART19-BE-01 trial. At study inclusion, the patient had palpable axillary lymphadenopathy, significant lymphocytosis and her bone marrow was infiltrated by 28% of CD19-positive but CD20-negative CLL cells. Fluorescent in-situ hybridization analysis revealed the presence of both 11q and 13q deletions in 70% and 76% of tumor cells, respectively. A computed tomography (CT) scan confirmed the presence of bilateral supraclavicular and axillary, celiac, and retroperitoneal lymphadenopathy (Fig. 1A and C). Despite her prior intolerance, the patient was advised to take ibrutinib 420 mg daily for 2 weeks (days −50 to −36) together with dietary recommendations and loperamide. She developed grade 2 diarrhea, which she tolerated relatively well and, as soon as her T-cells were collected, the medication was stopped. She has not received ibrutinib ever since that day. A total of 3.5 × 10^8^ T cells were harvested (97% purity), of which 70% were CD4^+^ and 29% were CD8^+^ on day −35. After 8 days of cell culture, 23% of cells became ARI-0001+ and were cryopreserved. As per protocol, fludarabine (30 mg/m^2^ per day) plus cyclophosphamide (300 mg/m^2^ per day) were administered on 3 consecutive days (−6, −5, and −4) and the patient received 1.0 × 10^6^ ARI-0001 cells/kg on day 0. Bilateral axillary lymphadenopathy was still palpable by physical examination on that day.

**Figure 2:**
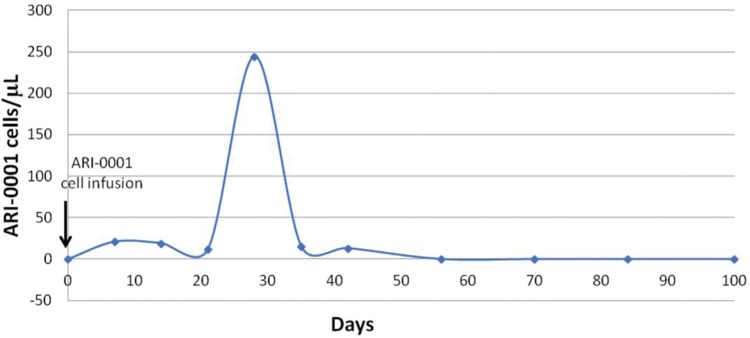
Concentration of ARI-0001 cells in peripheral blood over time (in absolute number per microliters).

The patient developed grade 1 cytokine release syndrome on day +1, which responded to antipyretics and antibiotics but never required advanced supportive measures nor tocilizumab. A gradual but robust in vivo ARI-0001+ cell expansion was observed over time, peaking at 30% of all circulating lymphocytes on day +28 (Fig. 2). On day +100, a bone marrow aspirate and biopsy were consistent with MRD-negative CR together with a complete absence of circulating or marrow CD19^+^ cells. Furthermore, a CT scan confirmed the almost complete disappearance of enlarged lymph nodes (Fig. 1B and D). The patient has received regular prophylactic immunoglobulin infusions and has had no significant infections so far. She remains in MRD-negative CR 1 year after the ARI-0001 cell infusion.

In conclusion, therapy for CLL has remarkably improved with the advent of novel targeted agents in the last 5 years. Unfortunately, there are patients who are either intolerant or refractory to these agents and have very limited therapeutic options. CART19 therapy could be feasible and efficacious in these patients, although it appears less effective compared with other B cell malignancies. Substantial preclinical work and at least 1 clinical trial suggest that prior therapy with ibrutinib may enhance its efficacy even though there is no formal proof for this statement.^4–6^ On the other hand, the optimal duration of ibrutinib treatment for this purpose is currently unknown. The only clinical trial currently addressing this issue requires a minimum of 6 months of therapy, which could be problematic in patients who are intolerant to the drug or in those with refractory disease. Our experience suggests that a shorter ibrutinib exposure may be sufficient for adequate immune activation of ARI-0001 cells in this setting but a prospective clinical trial would be needed to confirm this point. This trial is registered at clinicaltrials.gov (NCT03144583).
